# Colorimetric detection of magnesium (II) ions using tryptophan functionalized gold nanoparticles

**DOI:** 10.1038/s41598-017-04359-4

**Published:** 2017-06-21

**Authors:** Dae-Young Kim, Surendra Shinde, Gajanan Ghodake

**Affiliations:** Dongguk University-Seoul, Department of Biological and Environmental Science, College of Life Science and Biotechnology, Dongguk-ro, Ilsandong-gu, Goyang-si, Gyeonggi-do South Korea

## Abstract

The functional nanoparticles with specific molecular probe appear to be a promising approach for developing colorimetric nanosensor. In this work, we have synthesized tryptophan capped gold nanoparticles (AuNPs) and used to establish colorimetric detection of magnesium (Mg^2+^). The colorimetric response of the AuNPs toward Mg^2+^ was noticed with naked eyes, and spectral changes were monitored by using UV-Vis spectrophotometer. The detection response was rapid (less than 1 min), with a detection limit (LOD) about 0.2 µmol L^−1^. The proposed nanoprobe shows characteristic red-shift of the AuNPs at 620 nm and high selectivity for Mg^2+^ due to the binding affinity of the tryptophan with Mg^2+^. The real-time response of the UV-Vis spectrum was monitored at three different concentrations of Mg^2+^ (0.45, 0.50, and 0.55 µmol L^−1^). The AuNPs probe was suitable to provide a molecular platform for selective coordination with Mg^2+^ over Ca^2+^ ions, thus it could be facile to establish a practically viable sensing system. Furthermore, experimental results were confirmed to exhibit excellent linear curve for urine and serum samples spiked with Mg^2+^. Thus, this nanosensor is practically useful for the detection of Mg^2+^, without using expensive instruments, enzymes and/or DNA molecules.

## Introduction

Magnesium (Mg^2+^) is the second most common intracellular cation after potassium. Mg^2+^ acts as a cofactor for more than 300 enzymes, and also play an important role in controlling ion transport, structural stabilization of the proteins, enzymes, and nucleic acids^1^. Mg^2+^ ion remains chelated in chlorophyll via carboxyl groups of the photosynthetic apparatus, thus play a central role in chlorophyll structure and functions^2^. Magnesium deficiency can result in a variety of disorders including, hypocalcemia, hypokalemia, and neurological disorders^3^. Water hardness caused by Ca^2+^ and Mg^2+^ is a great concern in both domestic and industrial wastewater management since it involves in cerebrovascular disease and cardiovascular risk^4^. The measurement of Mg^2+^ have been accomplished using traditional methods, such as atomic absorption spectroscopy (AAS) and inductively coupled plasma-mass spectrometry (ICP-MS). However, involving expensive instrumentation and complex detection procedures, the previously reported methods demands upgradations^5, 6^.

Several other methods have been reported to detect Mg^2+^ includes null point titration^7^, magnesium-sensitive electrodes^8^, divalent cation ionophores^9^, and 31P NMR spectroscopy^10^. However, these fluorophore-based approaches frequently encounter a selectivity problem with both Mg^2+^ and Ca^2+^ ^11, 12^. Thus, there is wide scope for developing a selective detection, concentration determination, and intracellular imaging. Furthermore, interference from the Ca^2+^ is the most common hindrance in developing selective colorimetric sensor in diagnostic and therapeutic applications^13^. Thus, developing a potential colorimetric method based on rationally designed surface chemistry is desirable to trigger selective dispersion/aggregation states of the AuNPs^5, 14^. For strong chelating interactions with Mg^2+^, the surface chemistry of the AuNPs was established by using functional ligands of the tryptophan.

**Figure 1:**
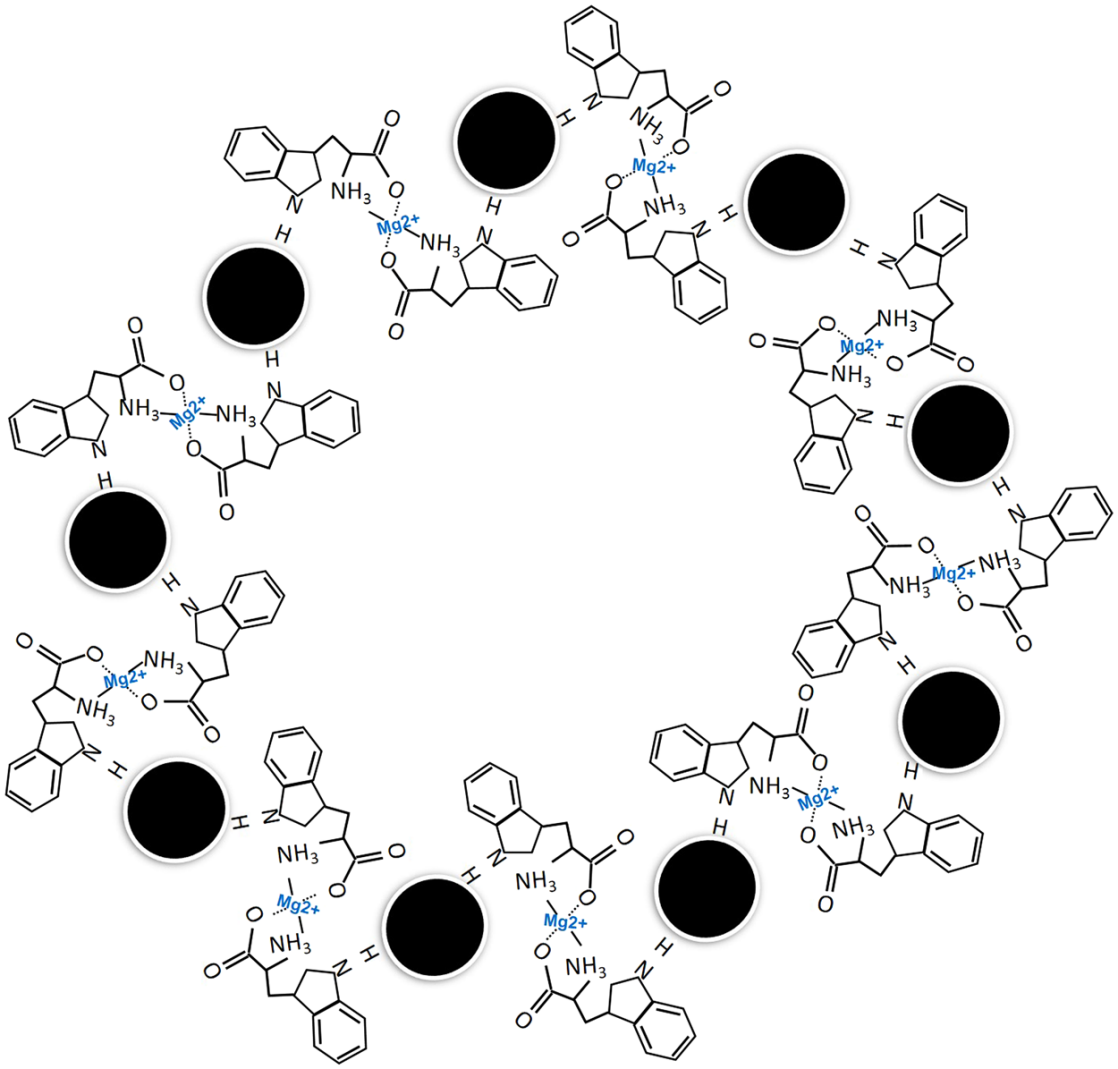
Proposed coordination mechanism of Mg^2+^ with tryptophan ligands.

This study demonstrates a facile, efficient, and sensitive colorimetric platform for the rapid monitoring of Mg^2+^. The binding of Mg^2+^ to the tryptophan ligands induces an apparent increase in absorbance at 620 nm. Here, tryptophan functioned AuNPs acts as an excellent chelating agent for Mg^2+^ via formation of the coordinated complex as illustrated in Fig. 1. Tryptophan amino acid was successfully used to functionalize the surface chemistry of the AuNPs and to cause the aggregation in the presence of Mg^2+^. This method seems advantageous to improve sensing of Mg^2+^ in terms of sensitivity and selectivity. Thus, we greatly anticipate finding wide applications in understanding physiological, pathological, and intracellular chemical events associated with Mg^2+^.

## Experimental

### Chemicals and reagents

Tryptophan and HAuCl_4_ were purchased from Sigma-Aldrich Chemicals. NaOH and NaCl were purchased from Dae Jung Chemicals Korea. MEM culture media was obtained from the Sigma Aldrich chemicals and used as an artificial serum. Standard solution of the metal ions includes, Mg^2+^, Hg^3+^, Co^3+^, As^+^, Li^+^, Cr^3+^, Ca^2+^, K^+^, and Mn^3+^ were obtained from the Kanto Chemical Company Ltd. Zn^3+^, Cd^2+^, and Pb^3+^ were obtained from the Nacalai Tesque Inc Kyoto, Japan. Stock solutions of 10 µM and 5 µM were prepared by the dilution of the standard stock solution (10.6 mM).

### Synthesis and characterization of the gold nanoparticles

A typical growth solution of the AuNPs was prepared as follows: 3-mL of the aqueous solutions tryptophan (6.25 mM) was added in different glass bottles containing diluted NaOH (5 mM). This solution was brought to 95 °C and the synthesis of the AuNPs was initiated by adding 1.0 mL of 20 mM HAuCl_4_. The AuNPs synthesis reaction was allowed to run for 48 hours at 95 °C. Centrifugation of the AuNPs solution was carried out at 10,000 rpm for 15 min to remove unbound tryptophan molecules and NaOH used in the synthesis. UV-Vis spectrophotometer (Optizen-2120) was used to record UV-Vis spectrum of the AuNPs in the wavelength range from 400 to 900 nm, with a resolution of 5 nm. Transmission electron microscope (TEM) images of the AuNPs were obtained from a Hitachi HF-3300. TEM samples were prepared by adding 100 μL of the AuNPs solution onto the 200-mesh Formvar-coated copper grid. X-ray diffraction (XRD) spectrum of the of the AuNPs thin film was measured by using Rigaku Ultima-IV System by operating at voltage 40 kV and current of 30 mA using Cu K radiation. FT-IR spectra of the centrifuged AuNPs was collected by using an infrared spectrometer (Thermo Electron Nicolet-6700).

### Determination of the standard solution of Mg^2+^

The measurement of the AuNPs sensitivity toward Mg^2+^ was carried out as follows: 300 μL of AuNPs stocking solution was added in water (total volume 1-mL) aqueous solution. The absorbance spectra of the AuNPs suspensions having different concentrations of Mg^2+^ (0.0, 0.10, 0.15, 0.2, 0.25, 0.3, 0.35, 0.4, and 0.45 µmol L^−1^) were measured respectively after 20 min of the incubation. Subsequently, concentration vs absorbance intensity at 620 nm was plotted to test the linear fit.

### Detection range of Mg^2+^

This report investigates the possibility of tuning the sensitivity of the AuNPs toward Mg^2+^ by using different concertations of the diluted NaOH (0.05, 0.1, and 0.2 mM). Typically, 300 μL of AuNPs was treated with 0.05 mM NaOH and total volume (1-mL) was adjusted by distilled water. The absorbance spectra of the AuNPs suspensions having different concentrations of Mg^2+^ (0.40, 0.50, 0.60, 0.70, 0.80, 0.90, and 1.0 µmol L^−1^) were measured after 20 min of the incubation. The same protocol was followed for quantification of Mg^2+^ in the range (0.80, 0.90, 0.1.0, 0.1.1, 1.2, 1.4, and 1.6 µmol L^−1^) at 0.1 mM NaOH. Furthermore, quantification of Mg^2+^ in a broad concentration range (1.2, 1.3, 1.4, 1.5, 1.6, 1.8, and 2.0 µmol L^−1^) was established at 0.2 mM NaOH.

### Real-time UV-Vis response of AuNPs toward Mg^2+^

UV-Vis spectral response of the AuNPs was detected at different time intervals after reaction with Mg^2+^. Typically, 300 μL of the AuNPs solution was suspended in water (total reaction volume was 1-mL). The UV-Vis spectrum of the AuNPs was acquired before exposure to Mg^2+^. Subsequently, AuNPs suspensions were treated with three different concertations of Mg^2+^ (0.45, 0.50, and 0.55 µmol L^−1^). The red-shift of the longitudinal and SPR band were observed for a long period of the time (20 to 360 min).

### Effect of pH and ionic strength

The effect of pH values on the AuNPs was studied as follows: 400 μL of the AuNPs stocking solution was suspended in water (total volume 1-mL) at different concertations of NaOH and HCl (0.0, 0.05, 0.1, 0.2, 0.3, 0.4 and 0.5 mM). These suspensions were placed inside the UV-Vis spectrophotometer for 10 min and the absorbance was measured at 530 nm. Then, 50 µL of 10 µmol L^−1^ stock Mg^2+^ solution was added to the respective NaOH-AuNPs suspensions. These suspensions were again placed inside the UV-Vis spectrophotometer for another 10 min and the absorbance was measured at 530 nm. The same protocol was followed to test the effect of NaCl (0.0, 1.0, 2.0, 4.0, 6.0, 8.0, and 10 mM), and CaCl_2_ (0.0, 0.1, 0.2, 0.4, 0.8, 1.2 and 1.6 mM) in identical conditions.

### Selectivity of the AuNPs

In addition, the selectivity of the AuNPs toward Mg^2+^ over other metal ions was investigated. The selectivity assay was conducted in water for Mg^2+^, Hg^3+^, Co^3+^, As^+^, Li^+^, Cr^3+^, Ca^2+^, K^+^, Mn^3+^, Zn^3+^, Cd^2+^, and Pb^3+^ at 200 ppb. The absorbance spectra of the AuNPs suspension were measured respective samples. These suspensions were placed inside the UV-Vis spectrophotometer for 20 min and the absorbance spectra of the suspensions were measured.

### Anti-interferential capability of the AuNPs

After testing the selectivity of the AuNPs toward Mg^2+^. At first, a mixture of the metal ions (Hg^3+^, Co^3+^, As^+^, Li^+^, Cr^3+^, Ca^2+^, K^+^, Mn^3+^, Zn^3+^, Cd^2+^, and Pb^3+^) was mixed with 600 µL of the AuNPs solution. Then the UV-Vis spectrum of the AuNPs was acquired in the presence of these metal ions. The UV-Vis spectrum was measured after adding 50 µL of 10 µmol L^−1^ Mg^2+^ to demonstrate the selectivity of the AuNPs.

### Determination of Mg^2+^ in urine and serum samples

UV-Vis spectral response of the AuNPs was collected after reacting urine and artificial serum samples containing Mg^2+^ ions. Typically, 300 μL of the AuNPs solution was treated with 0.5 mL urine and artificial serum samples. Finally, total volume about 1-mL was adjusted by adding distilled water. The UV-Vis spectrum of the AuNPs suspensions was acquired after 20 min of the incubation. Subsequently, concentration vs absorbance intensity at 620 nm was plotted. Unknown samples were monitored using standard graph prepared for urine and serum samples.

## Results and Discussion

### Characterization of the gold nanoparticles

**Figure 2:**
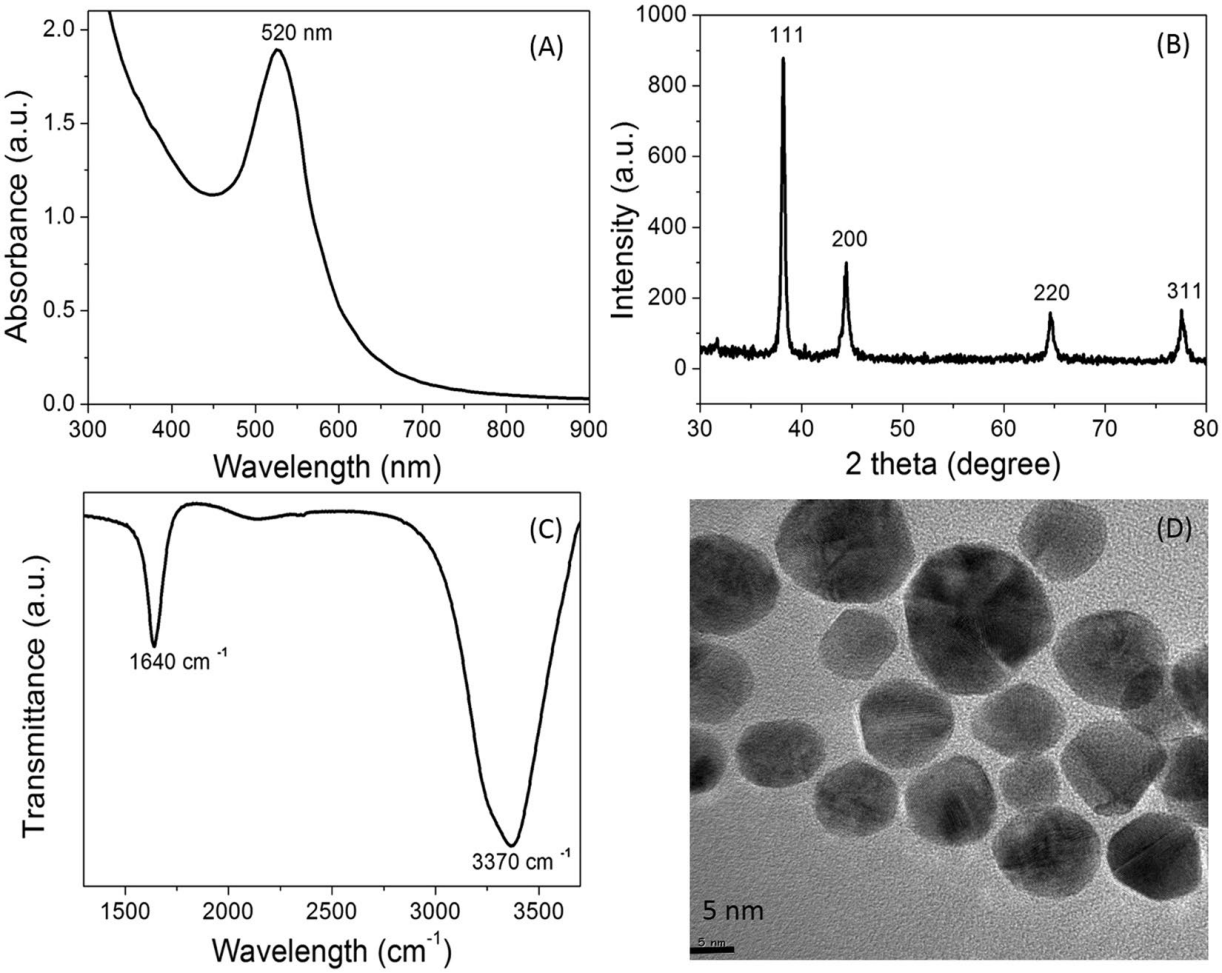
Characterization of the tryptophan capped AuNPs. (**A**) UV-Vis spectrum of the AuNPs, (**B**) XRD spectrum of the AuNPs, (**C**) FTIR spectrum of the AuNPs, (**D**), TEM imaging of the AuNPs.

The tryptophan capped AuNPs were used for characterization and application as a sensitive probe for Mg^2+^. The UV-Vis spectrum, size, shape, and structure of the AuNPs were examined by different techniques. Figure 2A shows a characteristic surface plasmon resonance (SPR) band of the as-prepared AuNPs located at 520 nm. The different diffraction peaks (111), (200), (220), and (311) was obtained by using JCPDS card: 004–0784 and it was confirmed that AuNPs were having cubic structure Fig. 2B. The mean crystallite size was calculated by using Scherrer equation D = Kλ/(β cos θ) and it was found about 16 nm. The size determined by XRD spectrum was in good agreement with TEM results. The lattice constant about 4.060 Ǻ was in agreement with the standard value (4.078 Ǻ) and similar value was reported for AuNPs^15^. The FT-IR spectrum of the centrifuged AuNPs was shown in Fig. 2C. The characteristic peak observed at 1640 cm^−1^ corresponds to the stretching vibrations of the C = O bond of the amide Fig. 2C. The FTIR spectra also showed a typical broad transmittance band at 3370 cm^−1^ from the OH stretching vibrations. The adsorptive interaction of the amino acid was reported with silica particles^16^. The AuNPs having spherical shape and narrow size-distribution with average size about 15 nm was observed by TEM. It can be seen that AuNPs were dispersed with few aggregates formed from the two or three nanoparticles in the Fig. 2D.

### Determination of the standard solution of Mg^2+^

**Figure 3:**
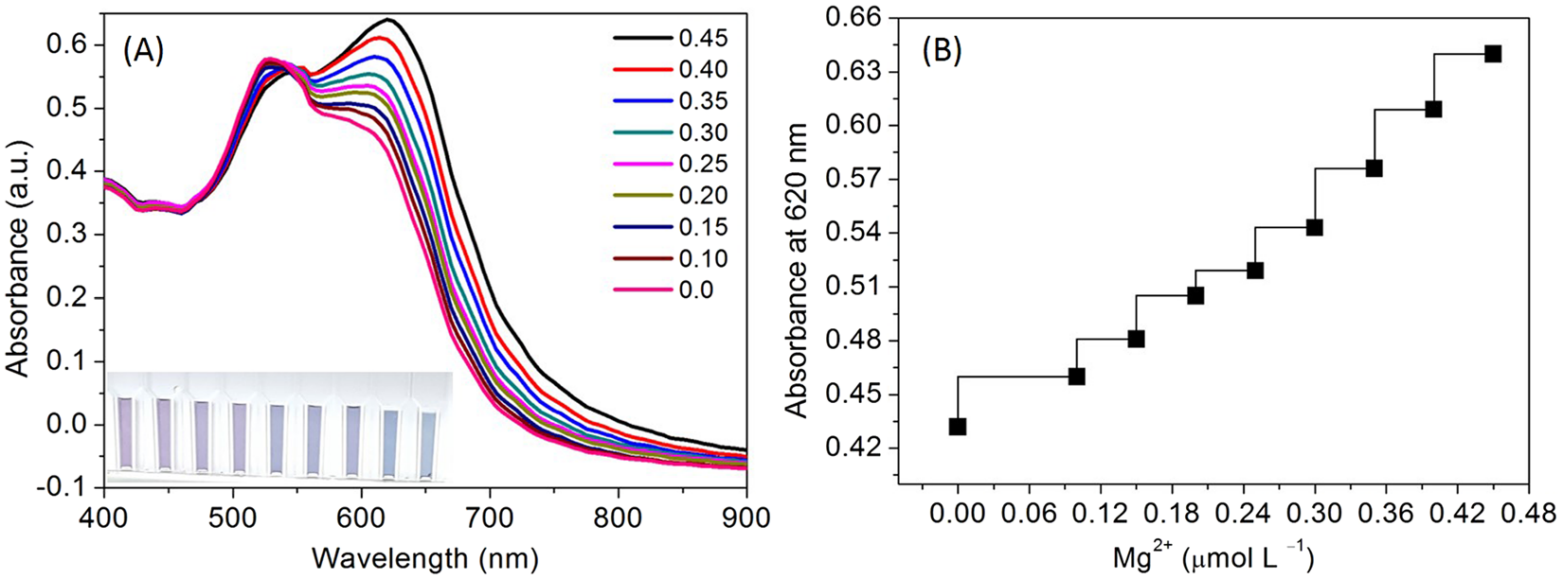
(**A**) Absorbance spectra of the AuNPs in the presence of increasing amounts of Mg^2+^ at ambient temperature; (**B**) The curve of absorbance intensity at 620 nm vs. Mg^2+^.

Sensing experiments were carried out by using centrifuged AuNPs having SPR band at 530 nm. As shown in Fig. 3A, the absorbance intensity of the AuNPs was increased gradually at 620 nm with the increasing concentration of Mg^2+^ (0.0, 0.10, 0.15, 0.2, 0.25, 0.3, 0.35, 0.4, and 0.45 µmol L^−1^). It was easy to demonstrate the assay for visual detection of Mg^2+^ based on the color change from purple to dark blue as shown in the inset of the Fig. 3A. A red-shift response of the longitudinal band emerged for the AuNPs exposed to Mg^2+^ from 0.35 to 0.45 µmol L^−1^ was prominent and stable at 620 nm. The red-shift of the longitudinal band and the characteristic spectral response attributes to the coupled plasmon absorbance of the AuNPs in close proximity. Furthermore, there was a linear relation between the relative absorbance intensity at 620 nm and the concentration of Mg^2+^ varying from 0.1 to 0.45 μmol L^−1^ with a detection limit of 0.2 µmol L^−1^ (Fig. 3B). It is significant to note that, the tryptophan functionalized AuNPs are appropriate to differentiate small variation in the Mg^2+^ concentration. The spectral changes resulted in a narrow concentration range of Mg^2+^, which meant that this method is reliable and sensitive enough to monitor Mg^2+^ content in water and diagnostic samples as compared with different methods summarized in Table 1. The different concentrations range of the Mg^2+^ (0, 1, 5, 10, 20 and 40 μM) was examined using Cys- DTT-AuNPs suspensions at room temperature^5^.

**Table 1:** Detection of Mg^2+^ contents in a reasonable range according to the literature values reported using different methods.

Method	Detection time	Detection range (µmol·L^−1^)	Detection limit (µmol·L^−1^)	Reference
Fluorescent	0.5 s	0 to 40	2.8	28
Fluorescent	—	0 to 10	5.1	29
Fluorescent	—	0 to 10	4.28	30
Colorimetric	—	0 to 70	0.1	31
Colorimetric	300 s	1 to 40	0.8	5
Colorimetric	60 s	0.1 to 2.0	0.2	This work

### Extending concentration range

**Figure 4:**
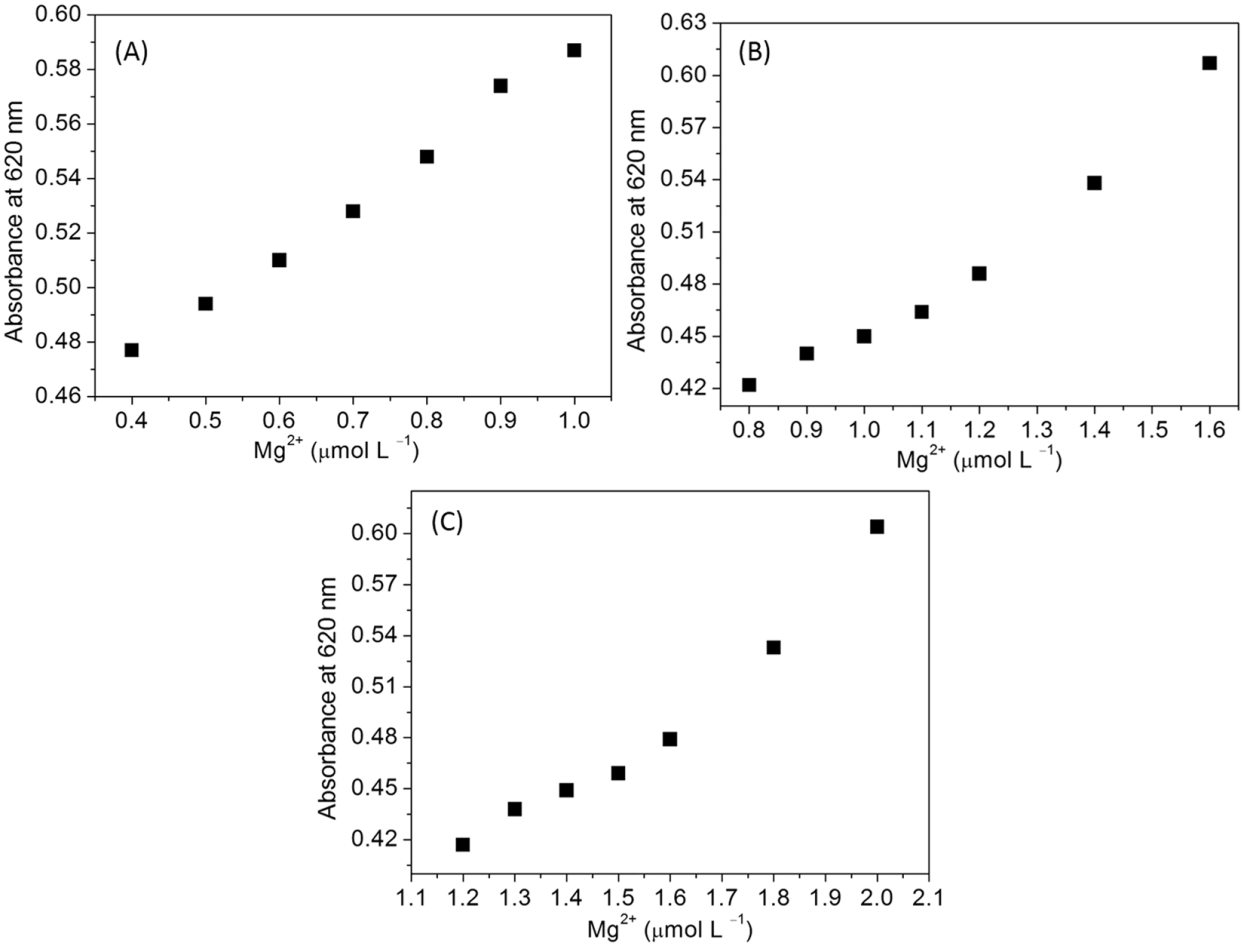
(**A**) The curve of the AuNPs absorbance intensity at 620 nm vs. Mg^2+^ in the range of 0.4 to 1.0 µmol L^−1^ at 0.05 mM NaOH, (**B**) The curve of the AuNPs absorbance intensity at 620 nm vs. Mg^2+^ in the range of 0.8 to 1.6 µmol L^−1^ at 0.1 mM NaOH, (**C**) The curve of the AuNPs absorbance intensity at 620 nm in the presence of increasing amounts of Mg^2+^ in the range of 1.2 to 2.0 µmol L^−1^ at 0.2 mM NaOH.

This sensing system has been explored to demonstrate the possibilities of testing a wide range and quantitative detection of Mg^2+^. The intensity of the absorbance was highly responsive toward Mg^2+^. Tryptophan capped AuNPs showed enhanced stability in diluted NaOH concentrations (0.05 to 0.2 mM). Thus, the absorbance enhancement was adjusted at different concentrations of NaOH (0.05, 0.1, and 0.2 mM). The AuNPs were made suitable for detecting and quantification of Mg^2+^ due to their pH sensitivity. A linear response for Mg^2+^ ions from 0.4 to 1.0 µmol L^−1^ was established by adding of 0.05 mM NaOH to the detection system (Fig. 4A). A linear response of the AuNPs toward Mg^2+^ in the range from 0.8 to 1.6 µmol L^−1^ at 0.1 mM NaOH (Fig. 4B). In addition, this assay abled probing a wide range of the Mg^2+^ (1.2 to 2.0 µmol L^−1^) at 0.2 mM NaOH (Fig. 4C), which may be attributed to the reducing intermolecular interactions of the Mg^2+^ coordination by deprotonation of the tryptophan ligands. Thus, the proposed method has potential in extending the detection range of Mg^2+^ from 0.1 to 2.0 µmol L^−1^ by adding diluted NaOH in the detection system. Thus, the linear responses of the AuNPs toward Mg^2+^ can be tuned using diluted NaOH, indicating the possibility to detect Mg^2+^ in a wide range of the concentrations. These results also suggest that the coordination of tryptophan with Mg^2+^ is also possible via the NH_2_ group. Thus, we propose tuning of the analytical probe is possible to widen the detection range by adding diluted NaOH in the detection system.

### Temporal response of the AuNPs toward Mg^2+^

**Figure 5:**
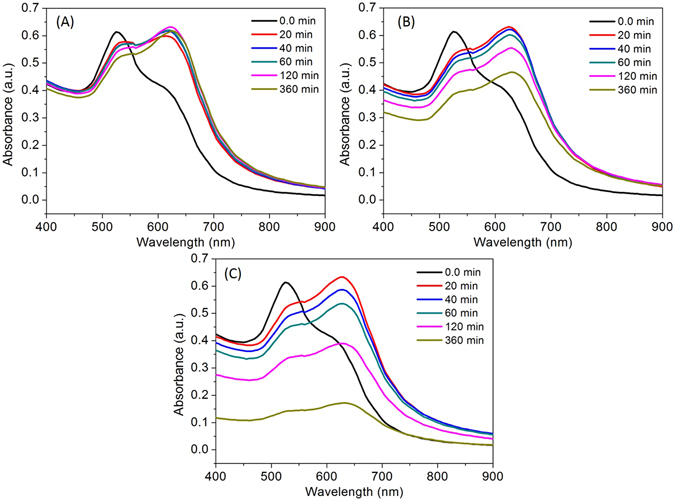
(**A**) Time course of the spectral response of the AuNPs at 0.45 µmol L^−1^ Mg^2+^, (**B**) Time course of the spectral response of the AuNPs at 0.50 µmol L^−1^ Mg^2+^, (**A**) Time course of the spectral response of the AuNPs at 0.55 µmol L^−1^ Mg^2+^.

Most of the colorimetric reports were based on the change in color and spectral response after addition of the target analyte. Real-time UV-Vis absorption spectroscopy was used to investigate the stability of the AuNP aggregates after coordination with Mg^2+^ and tryptophan ligands. The spectral response of the AuNPs was investigated at three different concentrations of Mg^2+^ (0.45, 0.5, and 0.55 µmol L^−1^) within the time frame of 20 to 360 min (Fig. 5). The red-shift of the longitudinal band was not affected after the reaction with Mg^2+^ (0.45 µmol L^−1^) even after incubation for 360 min indicates excellent stability of the AuNP aggregates (Fig. 5A). The fractal growth of the AuNP aggregates was detected by using a gradual decrease in the absorbance after reaction with Mg^2+^ (0.50 µmol L^−1^) (Fig. 5B). The absorbance of AuNPs was decreased significantly with Mg^2+^ at 0.55 µmol L^−1^, suggests the aggregative behavior of the AuNPs as observed in Fig. 5C. Thus, the intensity of the absorbance can be used to monitor aggregation of the AuNPs induced by Mg^2+^. Previously, similar observations was reported for self-assembled nanostructures originated from the plasmonic coupling and aggregation mechanism of the silver nanoparticles^17^.

### Effect of pH and ionic strength

**Figure 6:**
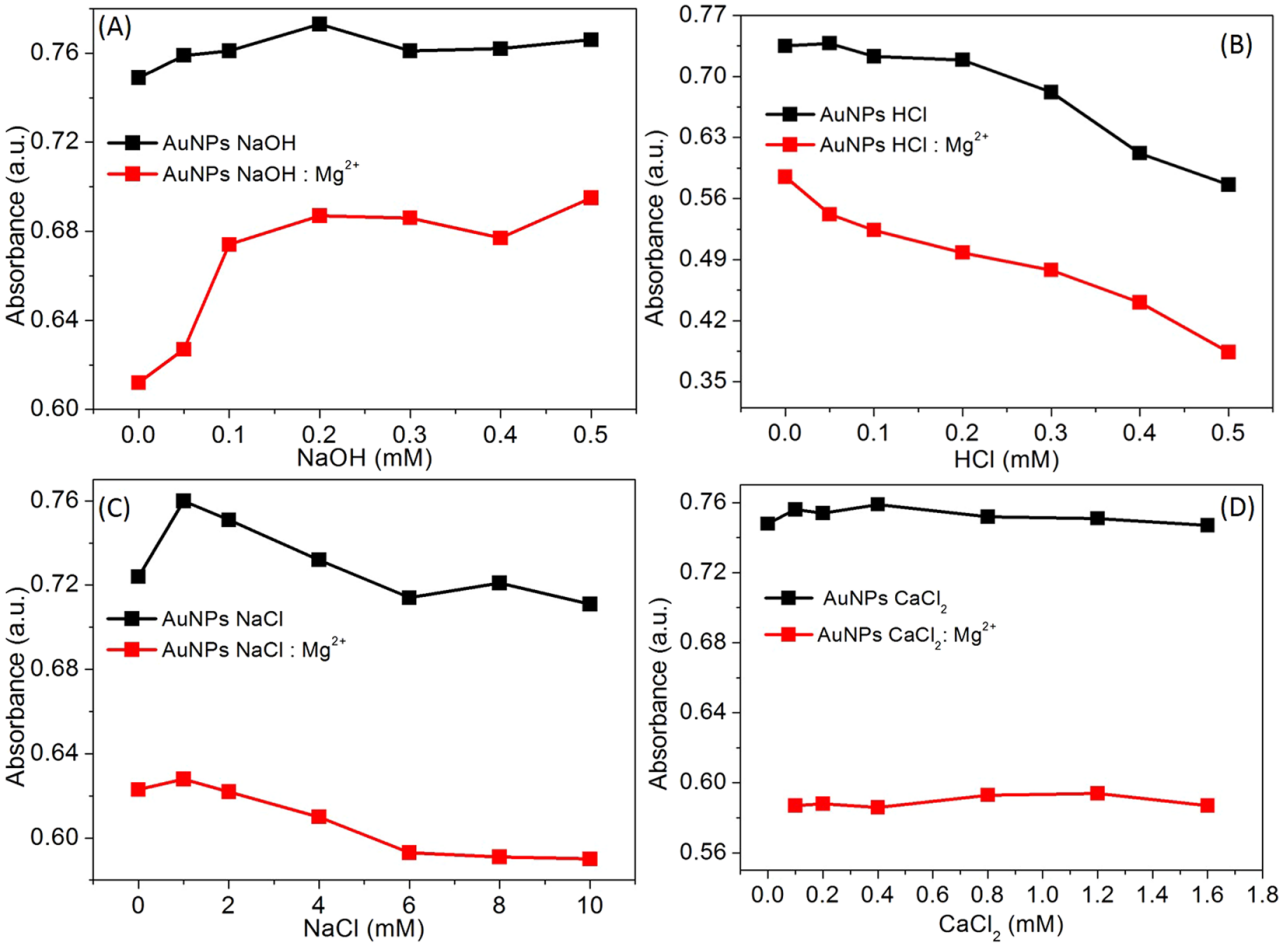
(**A**) Absorbance intensity of the AuNPs in the absence and presence of Mg^2+^ at different concentrations of NaOH, (**B**) Absorbance intensity of the AuNPs in the absence and presence of Mg^2+^ at different concentrations of HCl, (**C**) The effect of ionic strength on absorbance intensity in the absence and presence of Mg^2+^ (**B**) Absorbance intensity of the AuNPs in the absence and presence of Mg^2+^ at different concentrations of Ca^2+^. The absorbance intensity was recorded at 530 nm at ambient temperature.

The pH values of the AuNP suspensions has the tremendous impact on the detection of the target analyte. The Mg^2+^ sensing ability at different sensing conditions were investigated by measuring decrease in absorbance at 530 nm. The result shown in Fig. 6A suggest that AuNPs were stable within the NaOH range from 0.05 to 0.5 mM. However, its colorimetric response toward Mg^2+^ was decreased in the presence of diluted NaOH. The result observed with diluted HCl (0.05 to 0.5 mM) shows that the AuNPs were responsive to the acidic pH conditions. The absorbance intensity at 530 nm was significantly decreased in acidic conditions in the presence and the absence of Mg^2+^ ions (Fig. 6B). These results indicate that the tuning acidic conditions of the probe could be useful to enhance the sensitivity of the proposed nanosensor.

The ionic strength is also a significantly important parameter to detect target analyte^18, 19^. The effect of the ionic strength on the AuNPs was presented in the Fig. 6C. The absorbance intensity at 530 nm was decreased marginally in high ionic strength (6 to 10 mM), before and after the addition of Mg^2+^ (Fig. 6C). The addition of Mg^2+^ with the increasing concentration of NaCl, indicating the stability of the analytical platform at tested ionic strength.

### Selectivity and anti-interferential assay

High selectivity is significantly important to establish excellent colorimetric sensor. Thus, the selectivity of the AuNPs was examined by screening its response to individual metal ions (Mg^2+^, Hg^3+^, Co^3+^, As^+^, Li^+^, Cr^3+^, Ca^2+^, K^+^, Mn^3+^, Zn^3+^, Cd^2+^, and Pb^3+^) under identical conditions. The results showed that most of the other metal ions do not cause the colorimetric response of the AuNPs. However, the Mg^2+^ could increase the absorbance intensity of the AuNPs at 620 nm. According to the previous reports, tryptophan ligands may coordinate with metal ions^20, 21^.

**Figure 7:**
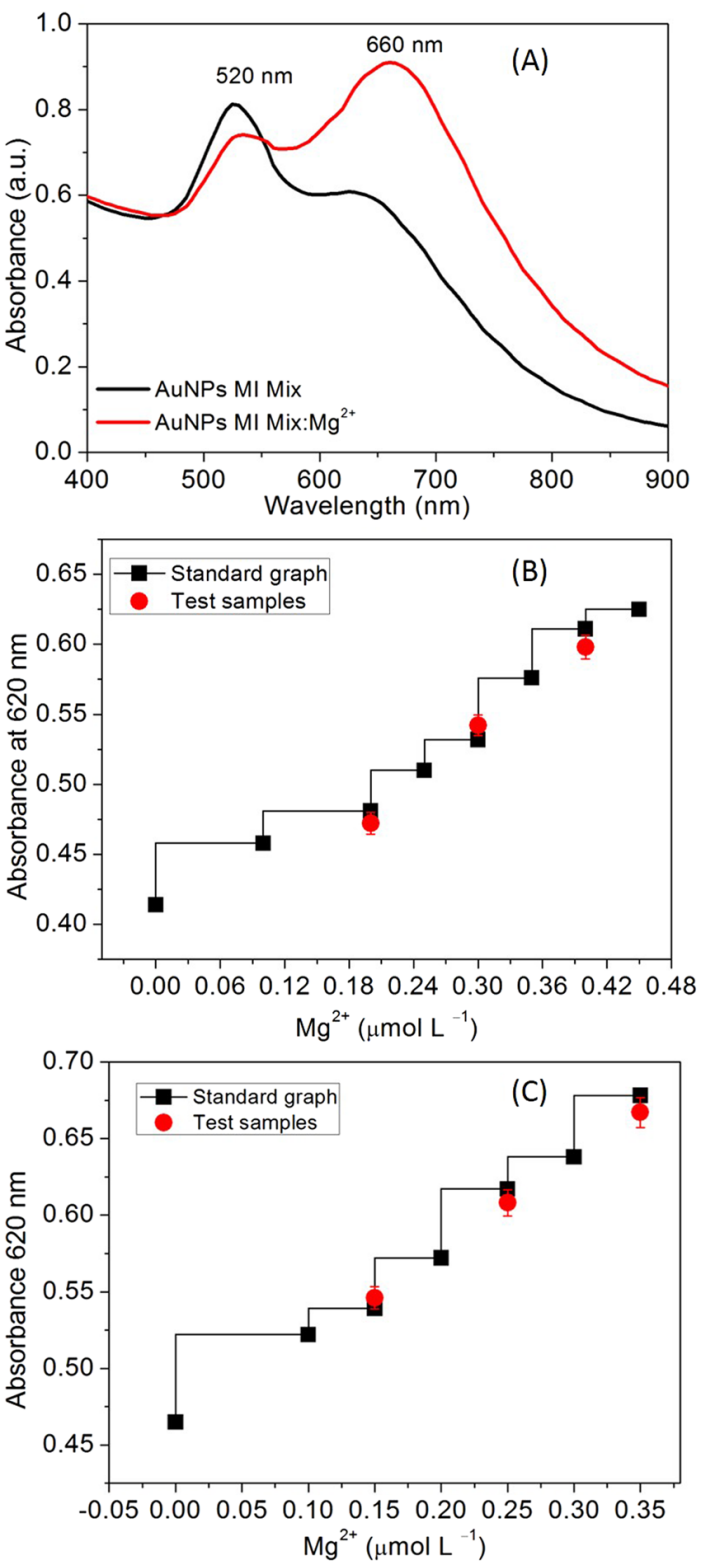
(**A**) UV-Vis response of the AuNPs upon the addition of Mg^2+^ (0.530 μmol L^−1^) to the solution containing a mixture of the metal ions, (**B**) The curve of the AuNPs absorbance intensity at 620 nm vs. urine samples spiked with Mg^2+^ was tested in the in the range of 0.1 to 0.45 µmol L^−1^, (**C**) The curve of the AuNPs absorbance intensity at 620 nm vs. artificial serum samples spiked with Mg^2+^ was tested in the range of 0.1 to 0.35 µmol L^−1^.

It was important to note that both Ca^2+^ and K^+^ do not compete with the tryptophan ligands of the AuNPs. Thus, the selectivity parameter was further investigated to ease the detection of Mg^2+^ in the presence of Ca^2+^. As we can see in the Fig. 6D, the absorbance intensity at 530 nm was not changed obviously before and after the addition of Mg^2+^ with the increasing concentration of Ca^2+^, suggesting vital selectivity of the analytical platform. In a case of Mg^2+^, tryptophan ligands take part in the interaction via both the carboxyl and amide groups as illustrated in the Fig. 1. The fluorescence resulted from the tryptotphan-26 was more heterogeneous and evident for Mg^2+^-bound complex with the troponin-I, however, Ca^2+^ showed no significant fluorescence^22^. Tridentate chelate forms, preferably after the interaction of Ca^2+^ with three oxygen atoms of the two carboxyl groups and the proton transfer by neighboring −COOH to −NH_2_ groups^23^. According to this, three amino acid residues are most suitable to play an important role in coordination of Ca^2+^ includes glutamic acid, γ-carboxyglutamate, and aspartic acid via Ca^2+^−carboxyl and Ca^2+^−carbonyl bond. Thus, the tryptophan ligands were not suitable to form a coordination complex with Ca^2+^ as it requires two carboxylic acid residues to bind and chelate them efficiently^24^. The interference from common metal ions was also investigated to expand the utility of this sensor. To demonstrate the ability to recognize Mg^2+^ in the presence of other competitive metal ions (Hg^3+^, Co^3+^, As^+^, Li^+^, Cr^3+^, Ca^2+^, K^+^, Mn^3+^, Zn^3+^, Cd^2+^, and Pb^3+^) was significantly important. Figure 7A, reveals that the mixture of the metal ions did affect the characteristic spectral response of the AuNPs toward Mg^2+^ and results a broad red-shift of the longitudinal band located at 660 nm. However, ability of the probe to recognize Mg^2+^ in the presence of other competitive metal ions could be demonstrated.

### Detection of Mg^2+^ in urine and serum samples

Renal magnesium wasting has been recognized in numerous conditions, including congenital defects, endocrine disorders (hyperaldosteronism and hyperparathyroidism), and exposure to the drugs (diuretics, cis-platinum, aminoglycoside antibiotics, and calcineurin inhibitors)^25^. A facile, rapid, and cost-effective methods are desired to diagnose impaired absorption in the intestines and excessive excretion of Mg^2+^ by the kidneys. This sensor was tested to reveal its applications in detecting renal function deterioration by measuring Mg^2+^ concentrations in urine and artificial serum samples. The similar linear curve was observed when the titrations were carried out in the presence of urine, suggesting that presence numerous metabolites in urine are not preventive for the detection of Mg^2+^ (Fig. 7B). The developed method also found appropriate for the measurement of Mg^2+^ content in the artificial serum (Fig. 7C). And it was found that these linear curves were suitable to determine the unknown concentration of Mg^2+^ from the urine and serum samples. The determined Mg^2+^ contents were at reasonable range in according to the literature values reported with other approaches Table 1. The observed minimum detection limit for Mg^2+^ in artificial serum was certainly lower than the diluted blood samples (~17 μM)^26^. Cerebral spinal fluid samples contain both Ca^2+^ and Mg^2+^ in mM level, thus proposed sensor is an excellent alternative for the detection of Mg^2+^, without the interference from the Ca^2+^ ions^5^. To our knowledge, no other small-molecular receptors were reported for Mg^2+^ in urine and serum samples. Selective detection of Mg^2+^ is appropriate to identify the significance of the Mg^2+^ in many physiological functions and pathological conditions^27^. Thus, results demonstrated reliability of the tryptophan capped AuNPs for detecting Mg^2+^ contents in real samples.

## Conclusions

In summary, a facile detection method based on absorbance probe AuNPs has been established, which allows highly sensitive determination of Mg^2+^. Functional AuNPs could be prepared easily and environmental friendly by mixing HAuCl_4_ to the tryptophan solution. Hence, this is the first report which explores sensing based on the tryptophan capped AuNPs for the analysis of Mg^2+^ content in a wide concentration range of Mg^2+^. The color change from purple to dark blue and well-defined red-shift appears within the fraction of a minute indicates coordination complex forms rapidly. The proposed design of a colorimetric assay where AuNPs acts as an indicator–receptor coupled with tryptophan ligands. The decrease in absorbance intensity of the AuNPs was significantly dependent on the concentration of Mg^2+^, thus the potential opportunity to prepare stable and unstable AuNP aggregates. This probe allows the detection of Mg^2+^ with high sensitivity and selectivity, without the interference from the competitive metal ions. Biologically relevant ions Ca^2+^ and K^+^ do not compete with AuNPs when it bounds with Mg^2+^. Furthermore, the proposed probe is suitable to detect Mg^2+^ content in water, urine, and serum samples.
